# *Candida albicans* colonization and dissemination from the murine gastrointestinal tract: the influence of morphology and Th17 immunity

**DOI:** 10.1111/cmi.12388

**Published:** 2014-11-25

**Authors:** Simon Vautier, Rebecca A Drummond, Kong Chen, Graeme I Murray, David Kadosh, Alistair J P Brown, Neil A R Gow, Donna M MacCallum, Jay K Kolls, Gordon D Brown

**Affiliations:** 1 Aberdeen Fungal Group, University of Aberdeen, Institute of Medical SciencesForesterhill, Aberdeen, AB25 2ZD, UK; 2 Department of Paediatrics, University of Pittsburgh School of MedicinePittsburgh, PA, 15224, USA; 3 Pathology, Division of Applied Medicine, University of AberdeenAberdeen, AB25 2ZD, UK; 4 Department of Microbiology and Immunology, University of Texas Health Science Center at San AntonioSan Antonio, TX, 78229-3900, USA

## Abstract

The ability of *Candida albicans* to cause disease is associated with its capacity to undergo morphological transition between yeast and filamentous forms, but the role of morphology in colonization and dissemination from the gastrointestinal (GI) tract remains poorly defined. To explore this, we made use of wild-type and morphological mutants of *C. albicans* in an established model of GI tract colonization, induced following antibiotic treatment of mice. Our data reveal that GI tract colonization favours the yeast form of *C. albicans*, that there is constitutive low level systemic dissemination in colonized mice that occurs irrespective of fungal morphology, and that colonization is not controlled by Th17 immunity in otherwise immunocompetent animals. These data provide new insights into the mechanisms of pathogenesis and commensalism of *C. albicans*, and have implications for our understanding of human disease.

## Introduction

*Candida albicans* is a polymorphic fungus which grows in both yeast and filamentous forms and resides as a commensal in humans, particularly within the gastrointestinal (GI) tract (Brown *et al*., 2012). However, *C. albicans* is also an opportunistic pathogen and one of the major aetiological agents of mucosal and systemic fungal infection (Brown *et al*., 2012). In susceptible individuals, systemic *C. albicans* infections are thought to arise from organisms in the GI tract; a hypothesis supported by data from both patients and animal models (Koh *et al*., 2008; Miranda *et al*., 2009). As filamentous forms predominate at sites of primary epithelial infection, morphogenic transition is thought to facilitate access of *C. albicans* to the bloodstream and subsequent systemic spread (Gow *et al*., 2012). Moreover, morphogenetic transition is essential for virulence of this pathogen as mutants locked in either the yeast (Lo *et al*., 1997) or filamentous (Murad *et al*., 2001) forms are highly attenuated in animal models of systemic disease.

Given the importance of mucosal barriers, considerable attention has been given to understanding the interactions between *C. albicans* and epithelial cells. These studies have generated evidence that hyphae, but not yeast, are responsible for damaging and triggering protective inflammatory responses in epithelial cells (Moyes *et al*., 2010; Wachtler *et al*., 2011). Moreover, hyphae can activate the inflammasome leading to IL-1β production and induction of the Th17 responses that are critical for protection at the mucosa (Hise *et al*., 2009; Joly *et al*., 2009; Cheng *et al*., 2011; Hernandez-Santos and Gaffen, 2012). Yet few studies have investigated the role of *C. albicans* morphology and host immunity during colonization of the GI tract *in vivo* (White *et al*., 2007; Pande *et al*., 2013), which is the focus of the studies presented herein.

## Results and discussion

**Figure 1:**
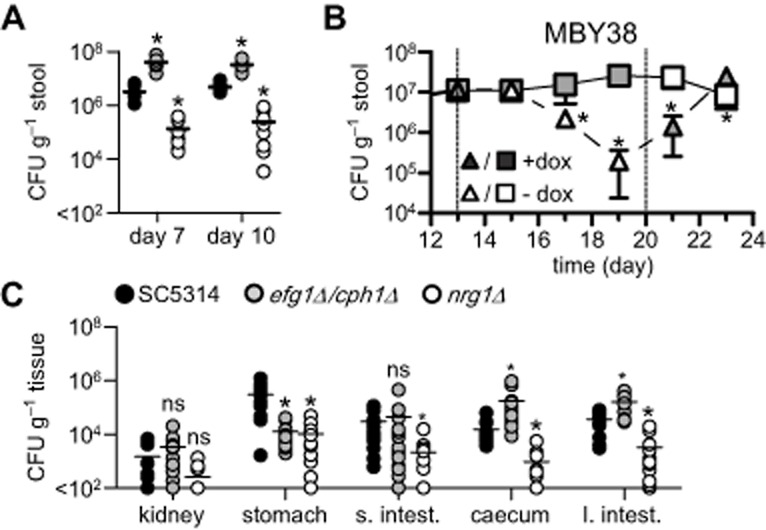
*Candida* *albicans* morphology influences colonization but not dissemination from the GI tract.A. Stool fungal burdens of 129Sv/Ev mice infected with wild-type (SC5314), yeast (*efg1*Δ/*cph1*Δ) and filamentous (*nrg1*Δ) *C. albicans* strains at day 7 and day 10 following infection (*n* = 10 per group).B. Stool fungal burdens of mice infected with MBY38 (*n* = 5 per group), following treatment with doxycycline, as indicated. The dotted lines indicate time points where doxycycline was administered or withdrawn.C. Tissue fungal burdens in the kidneys, stomach, small intestines (s. intest.), caecum and large intestines (l. intest.) at day 10 post infection with the fixed morphological mutants, as indicated (*n* = 14 per group). ns, not significant. *P < 0.05. See also Supporting Information Fig. S1.

To study the role of *C. albicans* morphology and host immunity during colonization of the GI tract, we made use of an established model whereby antibiotic-treated mice were colonized with *C. albicans*, following infection via their drinking water (Supporting Information Fig. S1A) (Vautier *et al*., 2012). Using this model, mice were infected with wild-type strains of *C. albicans* (SC5314 and CAI4) as well as strains carrying mutations locking them into the yeast (*efg1*Δ/*cph1*Δ) or filamentous forms (*nrg1*Δ). Differences in GI colonization were characterized by measuring stool fungal burdens at 7 and 10 days following infection (Fig. 1A). Over this time frame, the level of GI tract colonization remained constant for each strain, similar to our previous observations (Vautier *et al*., 2012) (Fig. 1A). Colonization with SC5314 was equivalent to that of CAI4, the parental strain from which the mutant strains were derived (Supporting Information Fig. S1B). Notably, when compared with wild-type *C. albicans*, mice infected with the yeast-locked *efg1*Δ/*cph1*Δ strain had higher fungal burdens in the GI tract while the filamentous-locked *nrg1*Δ strain colonized at lower levels (Fig. 1A). Similar results were obtained with other morphologically locked mutants, including yeast-locked *hgc1*Δ and filamentous *tup1*Δ (Supporting Information Fig. S1C). The lower colonization rates of the hyphal-locked mutants were not due to differences in inoculum, as we have previously shown GI tract colonization is not affected by the inoculum level (Vautier *et al*., 2012).

To further confirm that the filamentous form of *C. albicans* is not favoured in the GI tract, we utilized an inducible filamentous strain (MBY38; *tetO-UME6*) in a modified model of oral infection (Supporting Information Fig. S1D). This strain is a conditional *ume6* mutant, in which *UME6* is expressed in the absence of doxycycline, driving filamentous growth (Carlisle *et al*., 2009). We also infected mice with wild-type *Candida* to monitor for any effects of doxycycline itself on colonization levels. In the presence of doxycycline, MBY38 achieved similar colonization levels to SC5314, as measured by stool burdens (Fig. 1B and Supporting Information Fig. S1E). However, removal of doxycycline and induction of filamentous growth on day 13 post infection led to a rapid decline in colonization of this strain from the GI tract (Fig. 1B). Reintroduction of doxycycline at day 20 restored colonization levels. In contrast, GI colonization by SC5314 was unaffected by the presence or absence of doxycycline Supporting Information Fig. S1E).

We next investigated the ability of these mutants to colonize tissues of the GI tract by analysing fungal burdens at various sites on day 10 post infection (Fig. 1C). All strains were detected throughout the GI tract and generally reflected the levels found in the stools, i.e. *nrg1*Δ-infected animals had lower tissue burdens while *efg1*Δ/*cph1*Δ-infected animals had similar or higher tissue burdens when compared with SC5314 or CAI4 (Fig. 1C and Supporting Information Fig. S1F). Similarly, animals infected with the filamentous form of MBY38 had significantly lower tissue fungal burdens (Supporting Information Fig. S1G). In the stomach, however, both morphologically locked strains were present at lower levels compared with SC5314, suggesting that the ability to transition between morphologies is important for colonization of this tissue (Fig. 1C).

Although our results for these *C. albicans* strains reflect disparate observations made by several other groups (Bendel *et al*., 2003; White *et al*., 2007; Koh *et al*., 2008; Pierce and Kumamoto, 2012), it was possible that morphotype-dependent levels of colonization were being influenced through confounding effects of alterations in co-regulated genes in these strains. Thus, to determine the morphological preference in the GI tract in an unmodified strain, we enumerated the presence of yeast and hyphal forms of wild-type *C. albicans* in the stools, stomach and caecal contents of infected animals (Supporting Information Fig. S1H). In all samples, *C. albicans* was predominantly found as yeast. Thus, taken together, these results strongly suggest that GI tract colonization primarily favours the yeast form of *C. albicans*. In support of this conclusion, *C. albicans* was recently found to induce a novel yeast-like GUT (gastrointestinally induced transition) morphotype, following colonization of the GI tract (Pande *et al*., 2013). Why the yeast form is favoured is unclear, but may reflect host immunity to the filamentous forms, interaction with the microbiota and/or direct physical influences under conditions of flow within the GI tract.

Notably, we also found that *C. albicans* disseminated at low levels to the kidneys following GI colonization (Fig. 1C). Such dissemination has been reported previously (Kennedy and Volz, 1985; Samonis *et al*., 1990) and also occurred at equivalent levels with the mutant strains, which were highly attenuated in systemic infection models (Supporting Information Fig. S1I), as expected (Lo *et al*., 1997; Murad *et al*., 2001). Thus, these results show that dissemination from the GI tract is not dependent on morphological transition in *C. albicans*. How this dissemination occurs is unclear, as the intestinal epithelium is covered by a thick layer of mucus preventing fungal contact (de Repentigny *et al*., 2000; Johansson *et al*., 2011; Iliev *et al*., 2012). Indeed, we could not detect *C. albicans* cells in close proximity of the intestinal epithelium by histology [data not shown (Iliev *et al*., 2012; Vautier *et al*., 2012)]. Thus, transepithelial transport of these organisms must be mediated by indirect mechanisms, such as by lumen sampling dendritic cells (Rescigno *et al*., 2001) or M cell transcytosis (Owen *et al*., 1986; Rochereau *et al*., 2013). Such mechanisms could explain how *C. albicans* can disseminate from the GI tract, even in the absence of mucosal damage (White *et al*., 2007; Koh *et al*., 2008; Vautier *et al*., 2012). Importantly, dissemination from the GI tract does not result in systemic disease in immunologically competent animals (data not shown).

**Figure 2:**
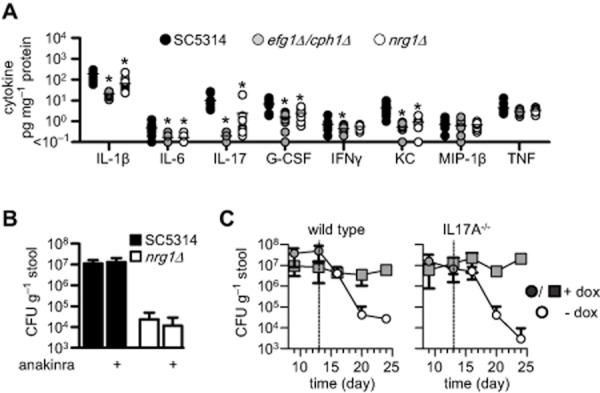
Immune responses during GI tract colonization.A. Selected cytokine levels in the stomachs of 129Sv/Ev mice at day 10 following infection with wild-type (SC5314), yeast-locked (*efg1*Δ/*cph1*Δ) and filamentous-locked (*nrg1*Δ) *C. albicans* strains (*n* = 10 per group).B. Day 6 stool fungal burdens of mice infected with wild-type (SC5314) and filamentous-locked (*nrg1*Δ) *C. albicans* strains, following daily treatment with IL-1RA (Anakinra), as indicated (*n* = 5 per group).C. Stool fungal burdens of C57BL/6 wild-type (*n* = 4 per group) or *Il17A*^−/−^ (*n* = 5 per group) mice infected with MBY38 in the presence or absence of doxycycline, as indicated. The dotted lines indicate time points where doxycycline was withdrawn. *P < 0.05. See also Supporting Information Fig. S2.

We next determined if the various strains of *C. albicans* differentially influenced host immunity by analysing cytokine levels in various tissues at day 10 post infection. However, we only detected altered cytokine levels in the stomachs of infected animals with the morphologically locked strains (Fig. 2A), which correlated with their reduced tissue fungal burden (see Fig. 1C). Such differences were not observed in any other tissue (Supporting Information Fig. S2A). This is consistent with our previous observations demonstrating preferential infection of the stomach in this model (Vautier *et al*., 2012) and the fact that that colonization is restricted to the lumen elsewhere in the GI tract (discussed above). Notably, the stomachs of animals colonized with the morphologically locked mutants had reduced levels of IL-1β, IL-6 and IL-17; all of which are involved in mediating Th17 responses (Hernandez-Santos and Gaffen, 2012).

Th17 immunity is required for controlling *C. albicans* infections at the mucosa, although the exact role of this response at different mucosal sites, especially in the GI tract, is controversial and poorly understood (Hernandez-Santos and Gaffen, 2012). We therefore explored the possibility that interfering with Th17 responses would alter GI tract colonization, particularly of filamentous forms. As IL-1β is essential for controlling systemic candidiasis (Vonk *et al*., 2006) and driving Th17 immunity (Hernandez-Santos and Gaffen, 2012), we first blocked the effects of IL-1β by daily administration of an IL-1 receptor antagonist (Anakinra) to mice and monitoring stool fungal burdens (Fig. 2B and data not shown). The i.p. administration of Anakinra has physiological effects in the intestines at the doses used in our experiments (Meinzer *et al*., 2012). Despite its importance in systemic models (Vonk *et al*., 2006), blocking the IL-1 receptor had no effect on colonization levels of either SC5314 or *nrg1*Δ. This suggests that IL-1 receptor signalling is not involved in controlling GI tract colonization.

We then looked at the role of IL-17-related responses, monitoring *C. albicans* colonization in the GI tract of both IL17A^−/−^ and IL17RA^−/−^ mice (Fig. 2C and Supporting Information Fig. S2B). For these experiments, we made use of our inducible strain (MBY38) to examine the effects on colonization by the filamentous morphotypes. Unexpectedly, we found no differences in *C. albicans* colonization in either mouse knockout strain. As before, induction of filamentous growth by withdrawal of doxycycline led to rapid reduction in the fungal burdens in the GI tract, but this was not significantly affected in the knockout mice. Thus, although we cannot exclude a role for IL-22 (De Luca *et al*., 2010), Th17 immunity does not appear to regulate GI colonization by *C. albicans*.

In conclusion, our data show that in immunocompetent animals the yeast form of *C. albicans* is favoured during colonization of the GI tract, from which there is a constitutive low level of systemic dissemination which is not dependent on morphological transition. Moreover, we show that colonization of the GI tract is not influenced by Th17 immunity, indicating that other mechanisms of control are occurring in these tissues. These data provide new insights into the mechanisms of pathogenesis and commensalism of *C. albicans*, and have implications for our understanding of human disease.

## Experimental procedures

### Mice and ethics statement

Eight to twelve week old female 129Sv/Ev, C57BL/6, *Il17a^−/−^* and *Il17ra^−/−^* mice on C57BL/6 background were bred and maintained at specific pathogen-free facilities at the University of Aberdeen and University of Pittsburgh. Mice were randomly allocated to groups and housed in individually ventilated cages, and provided with food and water *ad libitum*. All experimentation conformed to the terms and conditions of United Kingdom Home Office licenses for research on animals and the University of Aberdeen and University of Pittsburgh ethical review committees.

### *Candida albicans* strains, culture media and growth conditions

*Candida albicans* strains used in this study included SC5314 (wild type), CAI4 (wild type), MBY38 [*tetO-UME6* (Carlisle *et al*., 2009)], *efg1*Δ/*cph*Δ*1* (Lo *et al*., 1997), *nrg1*Δ (Murad *et al*., 2001), *hgc1*Δ (Zheng and Wang, 2004) and *tup1*Δ (Braun and Johnson, 1997). Strains were routinely grown and maintained on YPD agar (Sigma-Aldrich). For the MBY38 strain, plates contained 40 μg ml^−1^ of doxycycline. For inoculum preparation, a single colony was grown in Sabouraud broth (Oxoid) at 30°C for 24 h with shaking. Cells were washed twice in sterile phosphate-buffered saline (PBS) and counted using a haemocytometer. Inoculums of the filamentous strains, *nrg1*Δ and *tup1*Δ, were standardized to 1 × 10^7^ CFU ml^−1^ of SC5314 by quantifying protein concentration (Meyers *et al*., 1998).

### GI model

The GI model was performed essentially as described previously (see Supporting Information Fig. S1) (Vautier *et al*., 2012). To reduce commensal flora, mice were provided with sterile antibiotic water containing 2 mg ml^−1^ of streptomycin (Invitrogen), 2000 U ml^−1^ of penicillin (Invitrogen), 0.25 mg ml^−1^ of fluconazole (Enzo) for 3 days and then switched to water containing the same concentrations of streptomycin and penicillin for a further 24 h. Mice were then provided with sterile water containing 1 × 10^7^ CFU ml^−1^ of *C. albicans*, 2 mg ml^−1^ of streptomycin and 2000 U ml^−1^ of penicillin for 5 days. After *C. albicans* exposure, mice were maintained on sterile water containing 2 mg ml^−1^ of streptomycin, 2000 U ml^−1^ of penicillin and 0.2 mg ml^−1^ of gentamicin (Invitrogen). For strain MBY38, 2 mg ml^−1^ of doxycycline was added in addition to the drinking water as detailed in Supporting Information Fig. S1. To monitor colonization, stools were collected from individual mice and homogenized in 1 ml of PBS, serially diluted, and 25 μl of each dilution plated on YPD agar containing 0.01 mg ml^−1^ of vancomycin (Sigma) and 0.1 mg ml^−1^ of gentamicin. Plates were incubated overnight at 37°C under aerobic conditions and fungal levels determined by viable cell count. Mice were killed at the indicated time points post exposure to *C. albicans*. Kidney, stomach, small intestine, caecum and large intestine samples (harvested in that order) were washed three times with 1 ml of sterile PBS to remove gut contents. Tissue weights were determined, and samples were transferred into tubes containing 0.5 ml of PBS, 0.05% (v/v) Triton X-100 and complete mini EDTA-free protease inhibitor cocktail (Roche). The tissues were then homogenized, serially diluted and plated on YPD as above. Cell debris was removed from the remaining tissue homogenates by centrifugation at 15871 g for 15 min at 4°C and stored at −80°C for subsequent cytokine analysis.

### IL-1R blocking

The GI model was performed essentially as above, except mice were injected i.p. daily throughout the experiment with 50 mg kg^−1^ Kineret (Anakinra, UDG) (Sgroi *et al*., 2011) or PBS starting 2 days prior to infection.

### Cytokine analysis

Cytokine levels were measured using the Bio-Rad, Bio-Plex Pro™ Mouse 23-Plex kit and analysed on the Bio-Plex system using Bio-Plex Manager™ software as per manufacturer's instructions. Stored tissue sample supernatants were defrosted and centrifuged for 15 min at 15871 g at 4°C to remove debris. For each test, 50 μl of undiluted sample was used and cytokine concentrations were normalized to sample protein concentrations (BCA kit, Pierce).

### Yeast/hyphae counts

Ten microlitres of samples from homogenized stools, stomach and caecal contents were added to slides, coverslips applied and *Candida* cells counted under a microscope at 40× magnification. Four fields of view were counted per sample. The percentage of yeast/hyphae was then represented as a ratio of the total number of cells counted (between 50 and 100 for every field of view).

### Statistical analysis

The two-tailed student's *t*-test was used to compare two groups, while multiple groups analyses were performed using two-way analysis of variance (ANOVA). All data were analysed with GraphPad Prism software version 5.04.

## Supporting Information

Fig S1 *Candida albicans* morphology influences colonization but not dissemination from the GI tract. (A) Cartoon representation of the systemic experimental GI tract colonization model with sampling points (underlined). (B) Stool fungal burdens of 129Sv/Ev mice infected with *C. albicans* SC5314 and CAI4 at day 7 and day 10 following infection (*n* = 4 per group). (C) Stool fungal burdens of 129Sv/Ev mice infected with wild-type (SC5314), yeast (*hgc1*Δ) and filamentous (*tup1*Δ) *C. albicans* strains at day 9 following infection (*n* = 6 per group). (D) Cartoon representation of the modified GI tract colonization model with doxycycline (dox) and *C. albicans* MBY38. (E) Stool fungal burdens of 129Sv/Ev mice infected with SC5314, following treatment with doxycycline, as indicated (*n* = 5 per group). The dotted lines indicate the time intervals where doxycycline was administered or withdrawn. (F) Tissue fungal burdens in the kidneys, stomach, small intestines (s. intest.), caecum and large intestines (l. intest.) at day 10 post infection with the wild strains, as indicated (*n* = 4 per group). (G) Tissue fungal burdens in the kidneys, stomach, small intestines (s. intest.), caecum and large intestines (l. intest.) at day 20 post infection with SC5314 or MBY38, as indicated (*n* = 5 per group). (H) Percentage yeast morphology of *C. albicans* SC5314 and CAI4 in stools and in stomach and caecum contents at day 10 post infection. (I) Survival of mice following intravenous infection with (1 × 10^5^) wild-type (SC5314), yeast (*efg1*Δ/*cph1*Δ) or filamentous (*nrg1*Δ) *C. albicans* strains (*n* = 6 per group). **P* < 0.05.

Fig S2 Immune responses during GI tract colonization. (A) Selected cytokine levels in the caecum and large intestines of 129Sv/Ev mice at day 10 following infection with wild-type (SC5314), yeast-locked (*efg1*Δ/*cph1*Δ) and filamentous-locked (*nrg1*Δ) *C. albicans* strains, as indicated (*n* = 10 per group). (B) Stool fungal burdens of wild-type C57BL/6 (*n* = 6) or *Il17ra^−/−^* mice (*n* = 9) infected with MBY38, following treatment with or without doxycycline, as indicated.
