# Supplementary material for: Candida albicans colonization and dissemination from the murine gastrointestinal tract: the influence of morphology and Th17 immunity
Source: Cell Microbiol. 2014 Nov 25;17(4):445–50. doi: 10.1111/cmi.12388 (PMC4409086; doi:10.1111/cmi.12388)
Supplement: Fig S1 — Candida albicans morphology influences colonization but not dissemination from the GI tract. (A) Cartoon representation of the systemic experimental GI tract colonization model with sampling points (underlined). (B) Stool fungal burdens of 129Sv/Ev mice infected with C. albicans SC5314 and CAI4 at day 7 and day 10 following infection (n = 4 per group). (C) Stool fungal burdens of 129Sv/Ev mice infected with wild-type (SC5314), yeast (hgc1Δ) and filamentous (tup1Δ) C. albicans strains at day 9 following infection (n = 6 per group). (D) Cartoon representation of the modified GI tract colonization model with doxycycline (dox) and C. albicans MBY38. (E) Stool fungal burdens of 129Sv/Ev mice infected with SC5314, following treatment with doxycycline, as indicated (n = 5 per group). The dotted lines indicate the time intervals where doxycycline was administered or withdrawn. (F) Tissue fungal burdens in the kidneys, stomach, small intestines (s. intest.), caecum and large intestines (l. intest.) at day 10 post infection with the wild strains, as indicated (n = 4 per group). (G) Tissue fungal burdens in the kidneys, stomach, small intestines (s. intest.), caecum and large intestines (l. intest.) at day 20 post infection with SC5314 or MBY38, as indicated (n = 5 per group). (H) Percentage yeast morphology of C. albicans SC5314 and CAI4 in stools and in stomach and caecum contents at day 10 post infection. (I) Survival of mice following intravenous infection with (1 × 10^5^) wild-type (SC5314), yeast (efg1Δ/cph1Δ) or filamentous (nrg1Δ) C. albicans strains (n = 6 per group). *P < 0.05. [file cmi0017-0445-sd1.pptx]

## Slide 1
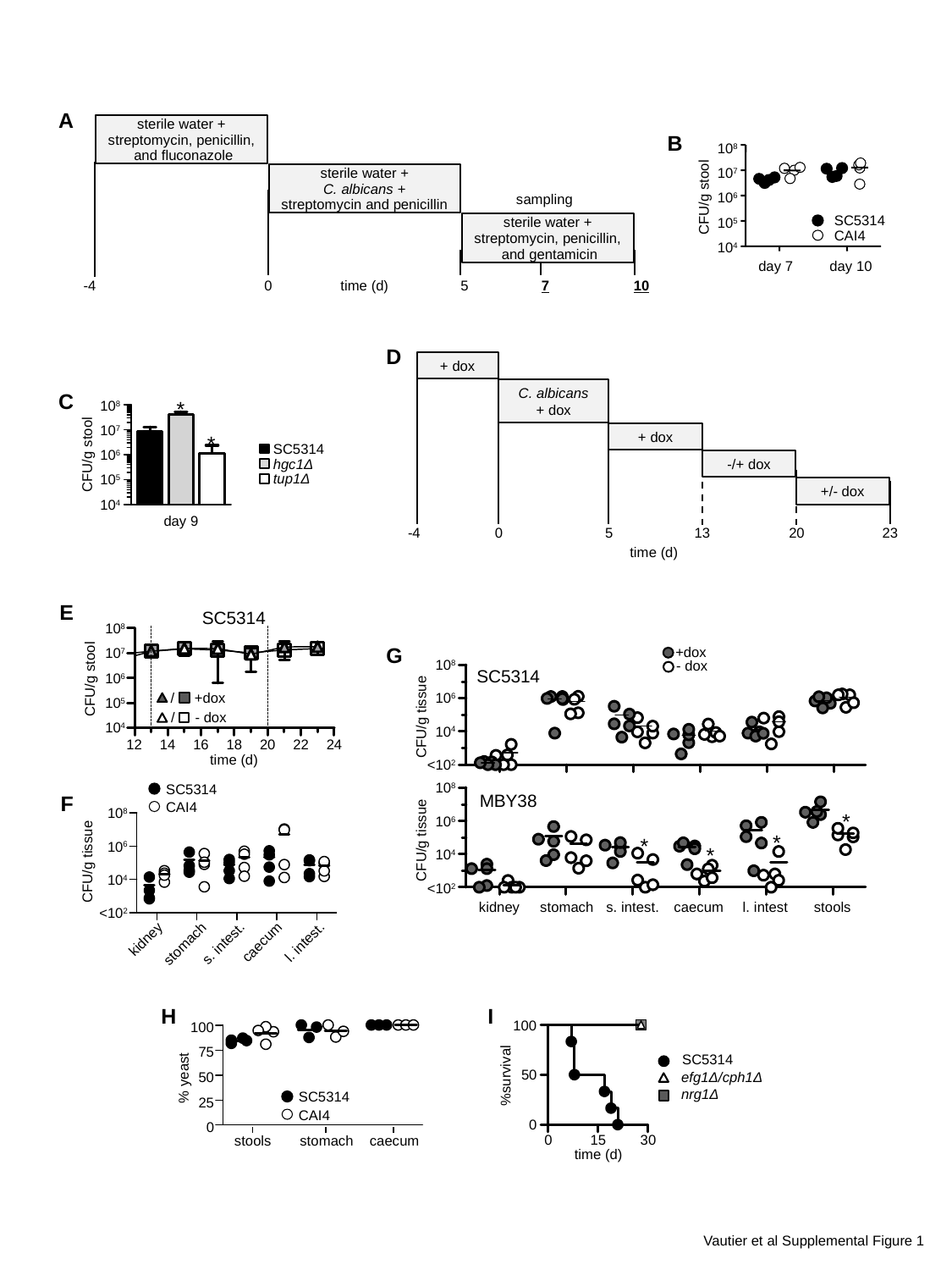

A
sterile water +
streptomycin, penicillin,
 and fluconazole
B
108
107
sterile water +
C. albicans +
streptomycin and penicillin
CFU/g stool
106
sampling
SC5314
105
sterile water +
streptomycin, penicillin,
 and gentamicin
CAI4
104
day 7
day 10
 -4 0 time (d) 5 7 10
D
+ dox
C. albicans
+ dox
C
*
108
107
+ dox
*
SC5314
CFU/g stool
106
hgc1Δ
-/+ dox
tup1Δ
105
+/- dox
104
day 9
-4
0
5
13
20
23
time (d)
E
SC5314
108
- dox
G
+dox
107
108
SC5314
106
CFU/g stool
/ +dox
106
105
CFU/g tissue
/ - dox
104
104
12
14
16
18
20
22
24
time (d)
<102
108
SC5314
MBY38
F
CAI4
108
*
106
*
CFU/g tissue
*
106
*
104
CFU/g tissue
104
<102
kidney
stomach
s. intest.
caecum
l. intest
stools
<102
kidney
l. intest.
caecum
s. intest.
stomach
H
I
100
100
75
SC5314
50
%survival
efg1Δ/cph1Δ
50
% yeast
nrg1Δ
SC5314
25
CAI4
0
0
0
15
30
stools
stomach
caecum
time (d)
Vautier et al Supplemental Figure 1
