# Supplementary material for: Candida albicans colonization and dissemination from the murine gastrointestinal tract: the influence of morphology and Th17 immunity
Source: Cell Microbiol. 2014 Nov 25;17(4):445–50. doi: 10.1111/cmi.12388 (PMC4409086; doi:10.1111/cmi.12388)
Supplement: Fig S2 — Immune responses during GI tract colonization. (A) Selected cytokine levels in the caecum and large intestines of 129Sv/Ev mice at day 10 following infection with wild-type (SC5314), yeast-locked (efg1Δ/cph1Δ) and filamentous-locked (nrg1Δ) C. albicans strains, as indicated (n = 10 per group). (B) Stool fungal burdens of wild-type C57BL/6 (n = 6) or Il17ra−/− mice (n = 9) infected with MBY38, following treatment with or without doxycycline, as indicated. [file cmi0017-0445-sd2.ppt]

## Slide 1
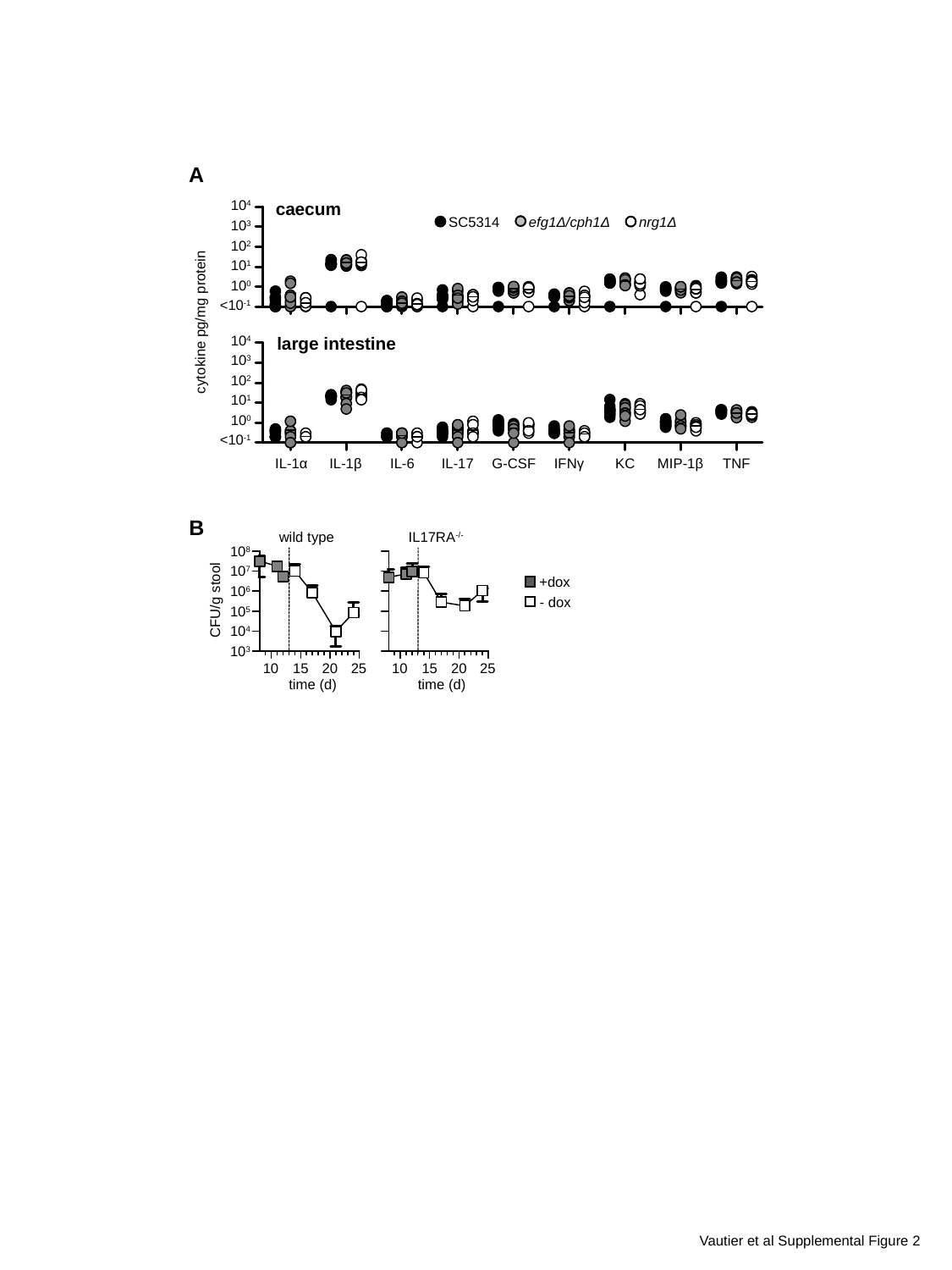

A
104
103
102
101
100
<10-1
caecum
cytokine pg/mg protein
104
103
102
101
100
<10-1
large intestine
IL-1α
IL-1β
IL-6
IL-17
G-CSF
IFNγ
KC
MIP-1β
TNF
SC5314
efg1Δ/cph1Δ
nrg1Δ
B
wild type
IL17RA-/-
108
107
 +dox
106
CFU/g stool
 - dox
105
104
103
10
15
20
25
10
15
20
25
time (d)
time (d)
Vautier et al Supplemental Figure 2
